# A Self-Powered Fast-Response Ultraviolet Detector of p–n Homojunction Assembled from Two ZnO-Based Nanowires

**DOI:** 10.1007/s40820-016-0112-6

**Published:** 2016-10-05

**Authors:** Yumei Wang, Ying Chen, Wanqiu Zhao, Longwei Ding, Li Wen, Haixia Li, Fan Jiang, Jun Su, Luying Li, Nishuang Liu, Yihua Gao

**Affiliations:** 1 Center for Nanoscale Characterization and Devices (CNCD), Wuhan National Laboratory for Optoelectronics (WNLO) & School of Physics, Huazhong University of Science and Technology (HUST), Luoyu Road 1037, Wuhan, 430074 People’s Republic of China; 2 Department of Mathematics and Physics, Zhengzhou University of Aeronautics, Wenyuan Road West 15, Zhengzhou, 450046 People’s Republic of China

**Keywords:** Zinc oxide, Micro/nano-assembling, p–n homojunction, Ultraviolet photodetector

## Abstract

**Abstract:**

Nowadays, fabrication of micro/nano-scale electronic devices with bottom-up approach is paid much research attention. Here, we provide a novel micro/nano-assembling method, which is accurate and efficient, especially suitable for the fabrication of micro/nano-scale electronic devices. Using this method, a self-powered ZnO/Sb-doped ZnO nanowire p–n homojunction ultraviolet detector (UVD) was fabricated, and the detailed photoelectric properties were tested. At a reverse bias of −0.1 V under UV light illumination, the photoresponse sensitivity of the UVD was 26.5 and the rise/decay time of the UVD was as short as 30 ms. The micro/nano-assembling method has wide potential applications in the fabrication of specific micro/nano-scale electronic devices.

**Graphical Abstract:**

A self-powered ZnO/Sb-doped ZnO nanowire p-n homojunction ultraviolet detector (UVD) was fabricated by using a novel micro/nano-assembling method with bottom-up approach. At reverse bias of −0.1 V under UV light illumination, the photoresponse sensitivity of the UVD was 26.5, and the rise time and decay time of the UVD were as short as 30 ms.
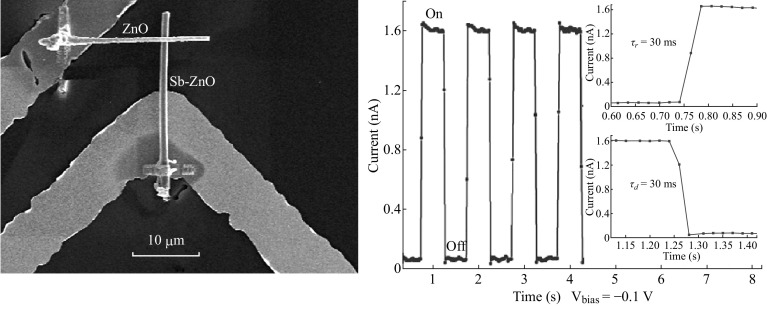

## Introduction

Due to the wide band gap of 3.37 eV, ZnO has attracted much attention for its potential applications in short-wavelength optoelectronic devices. Compared with another wide-band gap semiconductor of GaN, ZnO exhibits a lot of advantages, such as low-temperature synthesis, rich material source, low cost, and environmental friendliness. Since the first ZnO nanowire (NW) ultraviolet detector (UVD) was fabricated in 2002 [1], a lot of researches related to ZnO UVD have been reported. Generally, there are three types of ZnO UVDs from the point of view of different working principles: photoconductive UVD, Schottky junction UVD, and p–n junction UVD. Photoconductive UVD has a simple structure and a high gain [2–4], but its rise time and decay time are too long [5–10] due to the adsorption and desorption of oxygen molecules. Comparatively, Schottky junction UVD exhibits faster response speed [11, 12]. For example, Wang et al. reported a Schottky junction UVD by utilizing Schottky contact instead of Ohmic contact in device fabrication, and the reset time drastically reduced from ~417 to ~0.8 s [13].

ZnO p–n junction UVD, as a self-powered device, has attracted a great deal of research attention due to its fast response speed and high stability which are the unique advantages of p–n junction UVD [14–16]. However, the difficulties in fabrication of high-quality p-type ZnO hinder the applications of ZnO-based p–n junction UVD [17]. Most of ZnO p–n homojunctions are fabricated by two steps: growth of n-type ZnO NW section and p-type ZnO NW section [18–20]. Formation of ZnO p–n homojunction is much difficult due to the p-type doping difficulty of ZnO. Nevertheless, it is relatively easier to synthesize n-type ZnO and p-type ZnO NWs separately. Intrinsic ZnO has n-type conductivity, and group V elements are often used to form p-type doping of ZnO, such as nitrogen [21], phosphorus [20], arsenic [22], and antimony (Sb) [23–26]. Along with the development of micro/nano-assembling techniques, bottom-up fabrication of micro/nano-scale electronic devices comes true.

In this work, we demonstrate an efficient method to fabricate a self-powered ZnO p–n homojunction UVD, and high sensitivity and ultra-fast photoresponse speed were obtained. At a reverse bias of −0.1 V under UV light illumination, the ZnO p–n homojunction UVD has a sensitivity of 26.5, and the rise time *τ*_r_ (10–90 %) and decay time *τ*_d_ (90–10 %) of 30 ms. The size of UVD is very small and it can be integrated into a single chip to realize multifunction device. Our work presents an efficient and accurate way to build micro/nano-scale electronic devices with high performances.

## Experimental Section

### Synthesis of ZnO NWs and Sb-Doped ZnO (Sb–ZnO) NWs

Two NWs of ZnO and Sb–ZnO were synthesized via a simple chemical vapor deposition (CVD) method in a horizontal quartz tube furnace. A mixture of high-purity powders (weight ratio of ZnO:graphite = 1:1 for ZnO NWs; molar ratio of Zn:Sb_2_O_3_:graphite = 4:1:2 for Sb–ZnO NWs [24]) were used as the raw materials. For ZnO NWs, Au film with 3–5 nm thickness was firstly deposited on the Si substrate by a sputtering deposition method. In the growth process of ZnO, a mixed gas of 100 sccm Ar and 5 sccm O_2_ was used as the carrier gas and oxygen source, whereas 200 sccm Ar and 8 sccm O_2_ were used in the fabrication process of Sb–ZnO NWs. For ZnO NWs, the chamber was heated to 950 °C at a rate of 50 °C min^−1^ and lasted 40 min under 1 atm pressure, whereas it was heated to 930 °C and lasted 50 min for Sb–ZnO NWs. Then, the furnace was cooled down to room temperature naturally.

### Device Fabrication and Characterization

The Au/Ti electrodes with 10 μm finger spacing were fabricated by optical lithography and lift-off process on oxidized Si substrate (300-nm SiO_2_). The thicknesses of Au and Ti layers are 50 and 10 nm, respectively. The micro/nano-assembling process was performed in a dual-beam scanning electron microscope (SEM)/focused ion beam (FIB) microscope (FEI Quanta 3D FEG) equipped with nanomanipulator (Oxford Instruments OmniProbe 100) and gas injection system (GIS), and therefore the whole operating process can be monitored in real time. Firstly, one ZnO NW with suitable width and length was selected, and the tip of the nanomanipulator was handled carefully to make the tip contact with one side of the ZnO NW lightly. Secondly, the tip of the nanomanipulator and the ZnO NW were welded together by direct-written Pt deposition. Thirdly, the other side of the ZnO NW was cut off by the FIB. Finally, the ZnO NW was extracted and transferred it to anywhere we want.

The ZnO NW and another Sb–ZnO NW were transferred to the electrodes on chip. It is necessary to make one side of Sb–ZnO NW contact with the ZnO NW transferred before. The other sides of Sb–ZnO NW and intrinsic ZnO NW were welded on the Au electrodes by direct-written Pt deposition. Care was taken to avoid any contamination on the NWs’ surface on the chip.

The morphology, crystalline structure, and element distribution of the as-synthesized samples are characterized by SEM (FEI Nova Nano-SEM 450) and high-resolution transmission electron microscope (HRTEM, FEI Titan G2 60-300). The *I*–*V* characteristics and photoresponse of the devices were measured using an Agilent B2901A with the time resolution of 20 μs, a portable UV lamp (*λ* = 365 nm, 0.3 mW cm^−2^), and a function generator. All the electrical properties and photoelectric properties were tested at room temperature under atmospheric condition.

## Results and Discussion

**Fig. 1:**
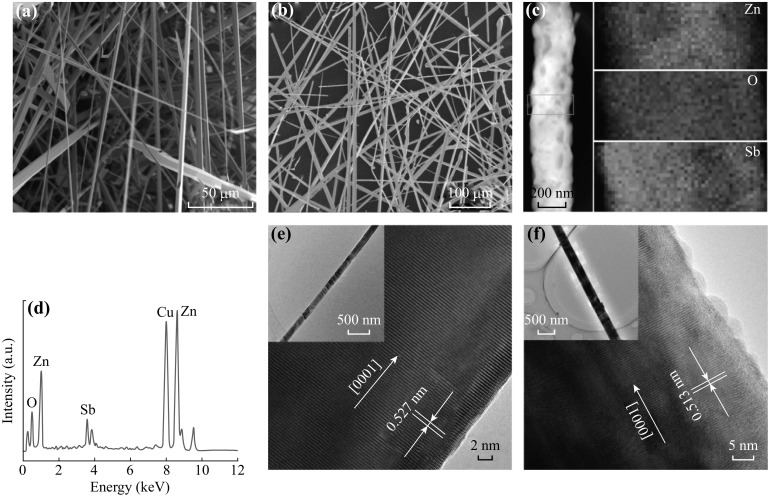
Characterization of ZnO and Sb–ZnO NWs. SEM images of **a** ZnO and **b** Sb–ZnO NWs. **c** EDS mapping images and **d** EDS spectrum of Sb–ZnO NW. TEM and HRTEM images of **e** ZnO NW and **f** Sb–ZnO NW

ZnO NWs and Sb–ZnO NWs were synthesized through a conventional CVD method. Sb–ZnO NWs have p-type characteristics due to the formation of Sb_Zn_–2V_Zn_ complex acceptor [23–26]. Typical SEM images of ZnO and Sb–ZnO NWs are shown in Fig. 1a, b. The ZnO and Sb–ZnO NWs are several micrometers in length, and 50 nm to 10 μm in diameter. The EDS spectrum (Fig. 1d) shows that the synthesized Sb–ZnO NWs are composed of O, Zn, and Sb elements. The content of Sb was estimated to be approximately 3 % (atom ratio). Figure 1c shows the elemental mapping images of Sb–ZnO NW, which reveals that the Sb element was doped into the ZnO NWs successfully and distributed uniformly. HRTEM images (Fig. 1e, f) of individual ZnO and Sb–ZnO NWs show clear lattice fringes, indicating that both of the NWs exhibit single-crystal structures and grow along the [0001] direction. However, the surface of Sb–ZnO NW is a rough amorphous layer (Fig. 1f). This phenomenon may be caused by the introduction of Sb^3+^ ion (radius 0.078 nm) that is larger than Zn^2+^ (0.074 nm), which leads to a large structural strain released by the rough surface [27].

**Fig. 2:**
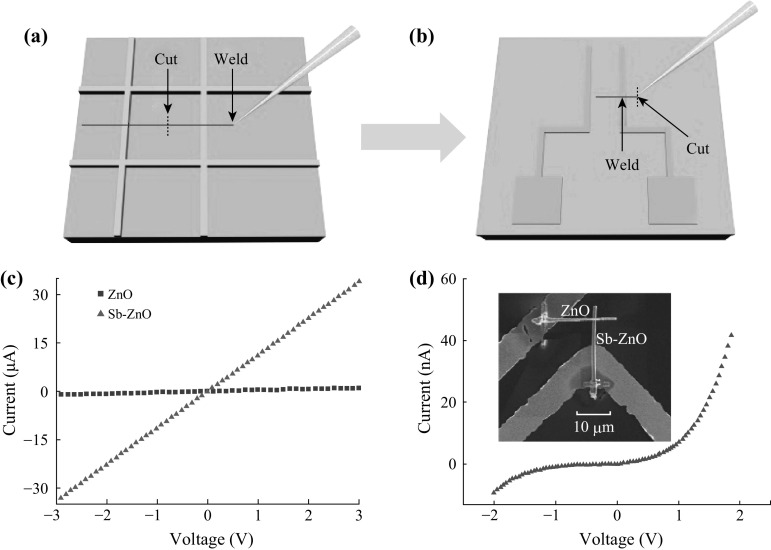
**a** and **b** Schematic diagrams showing the procedure of micro/nano-assembling. **c** *I*–*V* characteristics of a pure ZnO NW and a Sb–ZnO NW. The linear *I*–*V* curves indicate well Ohmic contacts. **d** *I*–*V* characteristics of p-type Sb–ZnO/n-type ZnO homojunction in dark. The *inset* SEM image in **d** shows the device structure

The ZnO/Sb–ZnO p–n homojunctions were fabricated in a dual-beam SEM/FIB microscope. Direct-written Pt deposition by FIB was used to prepare Pt electrodes. Even though the work function of Pt (~6.1 eV) is higher than that of pure ZnO (~5.1 eV), the contact electrodes of ZnO/Pt fabricated by Ga ion surface modification process and direct-written Pt deposition could still be Ohmic [28]. Figure 2c shows the *I*–*V* characteristics of the ZnO NW and Sb–ZnO NW. One can notice that both the *I*–*V* curves are linear, indicating good Ohmic contacts between ZnO (or Sb–ZnO) and Pt electrodes. The Sb–ZnO NW exhibits better conductivity than pure ZnO NW.

The fabrication processes of ZnO/Sb–ZnO p–n homojunction are schematically shown in Fig. 2a, b. The Ohmic ZnO/Pt contact and Sb–ZnO/Pt contact were made carefully by the same processes described above. The inset of Fig. 2d shows the SEM image of the device, where the ZnO NW and Sb–ZnO NW are connected. The ZnO and Sb–ZnO NWs have the diameters of ~500 nm and the lengths of ~20 μm. The *I*–*V* curve (Fig. 2d) of ZnO/Sb–ZnO p–n homojunction displays significant rectification characteristics under dark condition. The rectification ratio of the ZnO p–n homojunction diode is about 3.3 × 10^2^ at ±1 V, and the turn-on voltage is ~1 V.

**Fig. 3:**
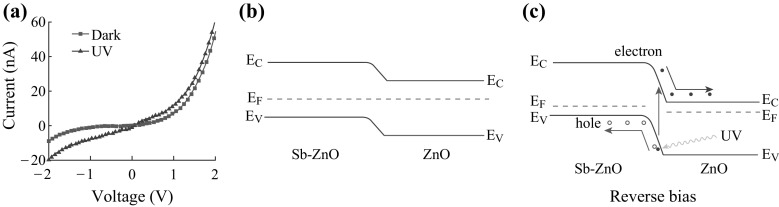
**a** *I*–*V* curves of ZnO/Sb–ZnO p–n homojunction in dark (*red*) and under 365 nm UV light illumination (*blue*). The energy band diagram of the p–n homojunction in **b** dark and under **c** UV light illumination at reverse bias

To investigate the photoelectric properties, the *I*–*V* characteristics of the ZnO/Sb–ZnO p–n homojunction were measured both in dark and upon 365 nm UV light illumination, as shown in Fig. 3a. The distance between the UV light and the device is fixed, and the power density of the UV light is about 0.3 mW cm^−2^. The UVD is responsive to the UV light significantly especially at reverse bias, which agrees with the behavior of the p–n junction photodetector. Figure 3b, c shows the energy band diagram of the p–n junction in dark and under UV light illumination at reverse bias, where the width of depletion layer and the barrier height will increase and weaken the dark current. When the contact area of ZnO and Sb–ZnO NW is exposed to UV light, the reverse bias will enhance the built-in electric field of the p–n junction. The photo-induced electrons and holes in the depletion layer will be swept away to the opposite direction quickly by the strong electric field, which will cause a large increase in photocurrent.

**Fig. 4:**
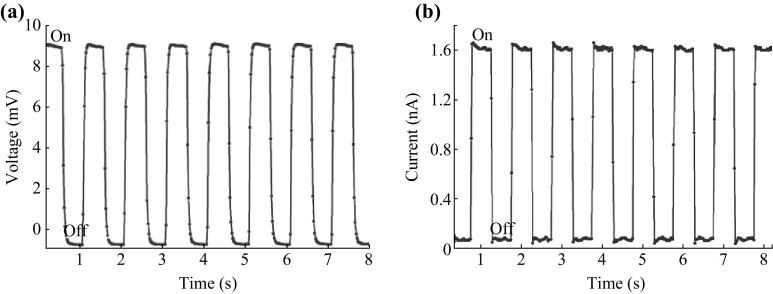
**a** Voltage–time curve of the p–n junction UVD at 0 V bias with 365 nm UV light on and off. **b** Current–time curve of the p–n junction UVD at a reverse bias of −0.1 V with 365 nm UV light on and off

Figure 4a, b shows the photoresponse of the UVD as a function of time when the UV illumination was switched on and off periodically, where the UV light was controlled by a function generator. At zero-voltage bias, the UVD worked as a self-powered device, and the open-circuit voltage *V*_oc_ is ~9 mV under UV light illumination. At a reverse bias of −0.1 V, the dark current is 60 pA and the photocurrent is 1.65 nA under UV light illumination. The sensitivity (defined as (*I*_light_ *−I*_dark_)/*I*_dark_) of the UVD is about 26.5. Both the voltage–time curve and the current–time curve show the stable and repetitive on/off cycles without significant noise. The ZnO/Sb–ZnO p–n junction UVD can work properly without external bias, which decreases the energy consumption or exhibits high sensitivity at certain reverse bias.

**Fig. 5:**
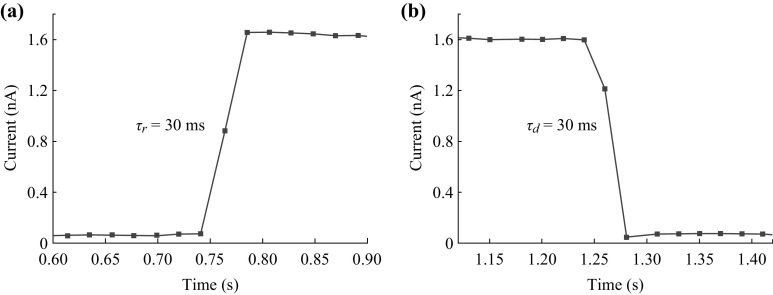
Enlarged current–time curves of the UVD of **a** the rise time and **b** the decay time, which were estimated to be 30 ms

Fast response speed is one of the outstanding advantages of p–n junction UVD. Figure 5 shows the enlarged current–time curve of the UVD at a reverse bias of −0.1 V with 365 nm UV light illumination. The rise time *τ*_r_ (10–90 %) and decay time *τ*_d_ (90–10 %) are estimated to be 30 ms, which are even shorter than those of the as-grown ZnO p–n homojunction UVDs [14, 29] and Schottky junction UVDs [11, 12, 30–32]. The fast-response mechanism is attributed to the p–n junction formed between ZnO and Sb–ZnO NWs, rather than the ZnO NW or Sb–ZnO NW photoconductor. The fast photoresponse performance is very stable and reproducible even in atmosphere environment.

**Fig. 6:**
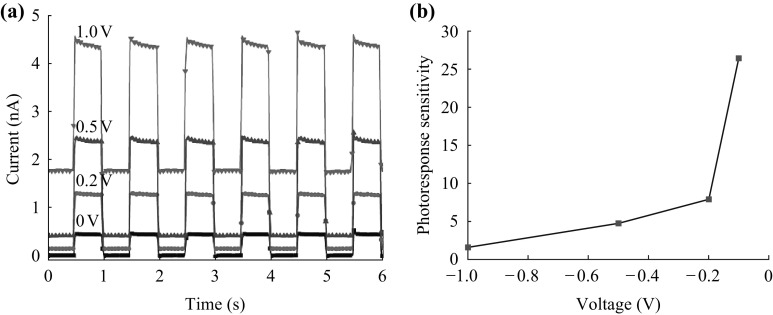
**a** Current–time curves of the p–n junction UVD at different reverse bias from −1.0 to 0 V with the 365 nm UV light on and off. **b** Photoresponse sensitivity of this UVD as a function of the applied reverse bias

Photoresponse of the UVD at different reverse bias from −1.0 to 0 V was also investigated. Figure 6a shows the current–time curves of the p–n junction UVD at different reverse bias with the 365 nm UV light on and off periodically. At zero-voltage bias, the short-circuit current *I*_sc_ is ~0.5 nA. The UVD works stable and reliable at different reverse bias, which implies wide potential applications. The photoresponse sensitivity of this UVD as a function of the applied reverse bias is shown in Fig. 6b. When higher extra reverse bias is applied, the dark current increases and the sensitivity decreases, which may be caused by the increasing leakage current.

The ZnO/Sb–ZnO p–n junction could be used as a UVD with fast response performance and high stability, or as a nano-scale photovoltaic cell. The micro/nano-assembling method has been proved to be an efficient and accurate way to build high-performance micro/nano-scale p–n junction UVD. The open-circuit voltage was improved using n-type ZnO and p-type ZnO with higher doping density, and it is much easier to control the doping density when the two kinds of NWs are synthesized separately. Moreover, with the help of the micro/nano-assembling method, we can freely combine semiconductor nanomaterials with different band gaps to build p–n junction photodetectors, which can be responsive to light of different wavelength ranges.

## Conclusion

In conclusion, we fabricated a ZnO/Sb–ZnO p–n junction UVD by a micro/nano-assembling method. The self-powered UVD exhibits high sensitivity and stability, and the rise time and decay time reach 30 ms. This micro/nano-assembling method provides an efficient and accurate way to fabricate micro/nano-scale electronic devices with potential applications.
